# Degradation of Butylated Hydroxyanisole by the Combined Use of Peroxymonosulfate and Ferrate(VI): Reaction Kinetics, Mechanism and Toxicity Evaluation

**DOI:** 10.3390/toxics12010054

**Published:** 2024-01-10

**Authors:** Peiduan Shi, Xin Yue, Xiaolei Teng, Ruijuan Qu, Ahmed Rady, Saleh Maodaa, Ahmed A. Allam, Zunyao Wang, Zongli Huo

**Affiliations:** 1 State Key Laboratory of Pollution Control and Resources Reuse, School of the Environment, Nanjing University, Nanjing 210023, China; peiduan_shi@163.com (P.S.); point_xinyue@163.com (X.Y.); quruijuan0404@nju.edu.cn (R.Q.); wangzy@nju.edu.cn (Z.W.); 2 Department of Zoology, College of Science, King Saud University, P.O. Box 2455, Riyadh 11451, Saudi Arabia; jajarem@ksu.edu.sa (A.R.); maodaa_28@yahoo.com (S.M.); 3 Department of Zoology, Faculty of Science, Beni-Suef University, Beni-Suef 65211, Egypt; allam1081981@yahoo.com; 4 Jiangsu Provincial Center for Disease Control and Prevention, No. 172 Jiangsu Road, Nanjing 210009, China

**Keywords:** ferrate, peroxymonosulfate, butylated hydroxyanisole, reaction pathways, toxicity evaluation

## Abstract

Butylated hydroxyanisole (BHA), a synthetic phenolic antioxidant (SPA), is now widely present in natural waters. To improve the degradation efficiency of BHA and reduce product toxicity, a combination of peroxymonosulfate (PMS) and Ferrate(VI) (Fe(VI)) was used in this study. We systematically investigated the reaction kinetics, mechanism and product toxicity in the degradation of BHA through the combined use of PMS and Fe(VI). The results showed that PMS and Fe(VI) have synergistic effects on the degradation of BHA. The effects of operational factors, including PMS dosage, pH and coexisting ions (Cl^−^, SO_4_^2−^, HCO_3_^−^, K^+^, NH_4_^+^ and Mg^2+^), and different water matrices were investigated through a series of kinetic experiments. When T = 25 °C, the initial pH was 8.0, the initial BHA concentration was 100 μM, the initial concentration ratio of [PMS]_0_:[Fe(VI)]_0_:[BHA]_0_ was 100:1:1 and the degradation rate could reach 92.4% within 30 min. Through liquid chromatography time-of-flight mass spectrometry (LC-TOF-MS) identification, it was determined that the oxidation pathway of BHA caused by PMS/Fe(VI) mainly includes hydroxylation, ring-opening and coupling reactions. Density functional theory (DFT) calculations indicated that ^•^OH was most likely to attack BHA and generate hydroxylated products. The comprehensive comparison of product toxicity results showed that the PMS/Fe(VI) system can effectively reduce the environmental risk of a reaction. This study contributes to the development of PMS/Fe(VI) for water treatment applications.

## 1. Introduction

Butylated hydroxyanisole (BHA), a synthetic phenolic antioxidant (SPA), is used as an additive and preservative in a wide range of food, packaging, pharmaceutical, cosmetic and other product categories [1]. Due to the broad application of BHA, it has been widely detected in water, with the highest detected concentrations reaching 17.4 ng/g and 135 ng/L in wastewater treatment plants in China and Spain, respectively [2,3]. The widespread presence of BHA has led to growing concerns about its impact on environmental health [4,5,6]. Toxicological studies have indicated that BHA may have adverse effects on developmental and reproductive systems, can exert estrogenic or antiestrogenic activities that interfere with the endocrine system, and can induce adipogenesis [7,8]. BHA is acutely and developmentally toxic to zebrafish, leading to organogenesis retardation in the early embryonic development of zebrafish [9]. Dietary exposure to BHA can also lead to pregastric papilloma and squamous cell carcinoma and induce pregastric tumors in rodents [10]. In addition, Some BHA metabolites may pose a health risk as they were reported to generate peroxides in mice and rats and may induce cellular DNA damage or have the capacity to be cancer initiators [2].

Current methods of BHA removal from water are mainly focused on photolysis, ozonation and chlorination. However, chlorination and ozonation are prone to produce toxic disinfection byproducts, and photoreaction devices are complex and expensive [11,12,13]. Advanced oxidation technology based on peroxymonosulfate (PMS), as a new oxidation technology, has attracted attention in the field of organic pollutant treatment due to its high redox potential, long half-life and wide application range [14,15,16]. The key to PMS-based oxidation is the formation of highly reactive species. The general methods for activating PMS include the use of external energy (such as heat, ultraviolet light radiation, ultrasound and microwave), chemical activators and transition metals or metal oxides [17,18,19,20,21]. However, SO_4_^•−^ is also susceptible to the influence of aqueous components (e.g., halides and natural organic matter), leading to the generation of some potentially toxic byproducts and unwanted oxidant consumption [22,23]. PMS-based advanced oxidation processes have been reported to produce more organochlorine acids, thereby increasing the toxicity of wastewater to mammalian cells [24].

In recent years, Fe(VI), as a multifunctional oxidant, has been used in water treatment [25]. It has been reported that Fe(VI)-based approaches have also been shown to be highly cost-effective for water remediation because they can be used more than once in the treatment procedure [26]. And the coupling of Fe(VI) and other oxidants (e.g., hydrogen peroxide (H_2_O_2_) and PMS) can be used to treat a wide range of micropollutants [27,28]. The combination of PMS and Fe(VI) has been reported to synergistically improve the removal of fluoroquinolones [28,29]. Wu et al. used Fe(VI) and PMS to degrade atrazine and showed that there was indeed a synergistic effect between Fe(VI) and PMS [30,31]. Therefore, the combination of PMS and Fe(VI) may be a valuable water treatment process. However, the degradation kinetics and potential mechanisms of PMS/Fe(VI) for the treatment of pollutants are not clear, especially regarding the toxicity changes in the intermediates during the reaction process.

Therefore, this study presents a systematic investigation regarding the feasibility of BHA degradation through the combined use of PMS and Fe(VI). The BHA degradation efficiencies of PMS alone, Fe(VI) alone and PMS/Fe(VI) were compared. In addition, the effects of solution pH, common inorganic ions and different water matrices on the removal rate were examined. The main active species of the reaction system were identified by EPR tests, and this result was correlated with theoretical calculations. Thermodynamic calculations demonstrated a possible synergistic reaction between PMS and Fe(VI). Detailed comparative analyses of the type, content and toxicity of the degradation products in the PMS/Fe(VI) and single-PMS systems were carried out with liquid chromatography–tandem mass spectrometry (LCMS/MS). This study provides new insights into the degradation of organic pollutants by PMS in combination with Fe(VI).

## 2. Materials and Methods

### 2.1. Chemicals and Water Samples

BHA, containing 90% 3-BHA and 10% 2-BHA, was obtained from Hebei Jijie Biotechnology Co., Ltd. (Hebei, China). Solid potassium ferrate (K_2_FeO_4_, >98%) was chemically synthesized by the wet method [32]. 5,5-dimethyl-1-pyrroline-N-oxide (DMPO, purity >99%) was purchased from Aladdin Chemical Co., Ltd. (Shanghai, China). Other chemicals used in the experiments to determine the influence of water chemistry, including PMS (>99%), NaCl (>99%), Na_2_SO_4_ (>98%), Na_2_S_2_O_3_ (>99%), NaHCO_3_ (>99%), K_2_SO_4_ (>99%), (NH_4_)_2_SO_4_ (>99%) and MgSO_4_ (>99%), were obtained from Aladdin Chemistry Co., Ltd. (Shanghai, China) and used directly without further purification.

Tap water (TW), synthetic wastewater (SW), secondary clarifier effluent (SCE) and river water (RW) were used to examine the effect of water matrices on PMS/Fe(VI). Synthetic wastewater was prepared from moderately hard freshwater according to the composition of local industrial wastewater, which contains NaCl (1000 mg L^−1^), citric acid (50 mg L^−1^), ascorbic acid (30 mg L^−1^), D(+)-sucrose (100 mg L^−1^) and Na_2_HPO_4_ (230 mg L^−1^). Secondary clarifier effluent and river water were collected from the Wulongkou sewage treatment plant (Zhengzhou, China) and Jiuxiang River (Nanjing, China), respectively. The main physicochemical parameters of the four water samples are listed in Table S1.

### 2.2. Experimental Procedures

The oxidative degradation experiments were conducted in 50 mL conical flasks. Then, a 100 µM BHA solution and borate buffer of a certain pH were added. Next, the conical flasks were placed on magnetic stirrers, undergoing rapid stirring to achieve a uniform reaction. The reaction was started when a certain amount of oxidant was added. In total, 1 mL of the reaction solution was removed and put into a 2 mL plastic centrifuge tube preadded with 200 µL of methanol and 100 µL of 0.2 M Na_2_S_2_O_3_ to terminate the reaction. The reaction temperature was 25 °C, and all experiments were performed in triplicate.

### 2.3. Analytical Methods

The BHA content was detected by means of a PerkinElmer liquid chromatograph (USA), the Flexar model, under a UV detector. The detailed HPLC setups can be found in Text S1. Possible intermediates in the reaction system were detected using an Agilent 1260 infinity HPLC system tandem. The detailed LC-MS/MS setup can be found in Text S2. The reactive oxygen species were detected by an electron paramagnetic resonance spectrometer (EPR; MiniScope MS 5000, Freiberg Instruments, Germany).

### 2.4. Theory Calculations

Density functional theory calculations were run on Gaussian 09 software. And the theory level used for the calculation was B3LYP/6-311G**. The geometric optimization and frequency calculations of reaction types, including reactants (REs), transition states (TSs) and products (PRs), were carried out. The Gibbs free energy (G) was obtained from the Gaussian output file [33,34,35,36]. The transition state had only one imaginary frequency, which was further verified by the intrinsic reaction coordinate (IRC). By contrast, a fully optimized configuration of the reactants and products had no imaginary frequency. To study the aqueous phase reaction, the Solvation Density Model was used to consider the solvent effect.

### 2.5. Toxicity Evaluation

The acute toxicity of BHA and its reaction intermediates to fish, Daphnia and green algae was evaluated using ECOSAR software [37]. The software has been approved by the Organization for Economic Cooperation and Development (OECD), the Environmental Protection Agency of the United States (US EPA) and the European Union (EU).

## 3. Results and Discussion

### 3.1. Degradation of BHA

**Figure 1:**
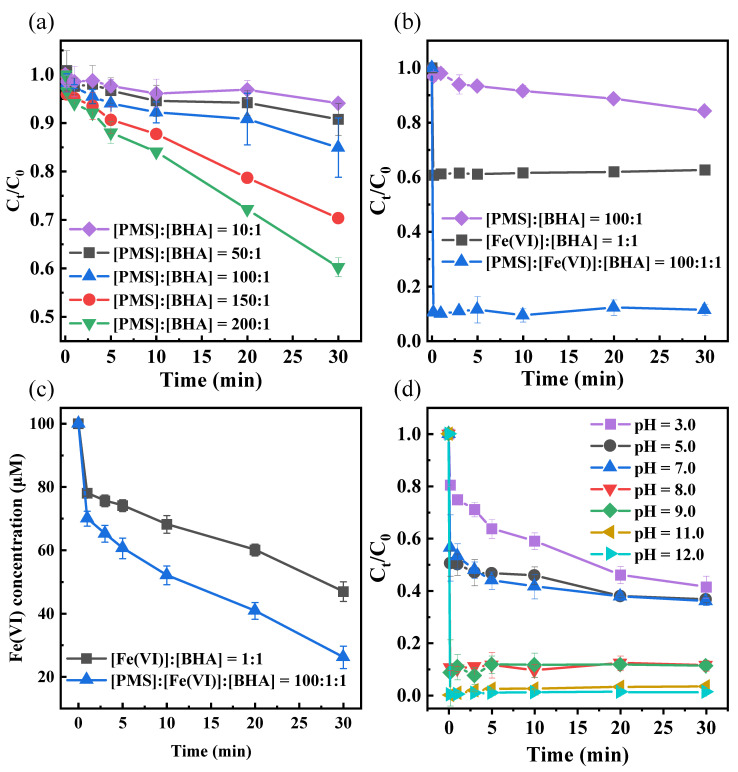
(**a**) Effect of PMS dosage on the degradation of BHA. Experimental conditions: [BHA]_0_ = 100 μM, pH = 8.0 and T = 25 °C. (**b**) Degradation of BHA by different processes. Experimental conditions: [BHA]_0_ = 100 μM, pH = 8.0 BS T = 25 °C. (**c**) Change in Fe(VI) concentration during degradation of BHA by Fe(VI) and PMS/Fe(VI). Experimental conditions: [BHA]_0_ = 100 μM, pH = 8.0 and T = 25 °C. (**d**) Effect of different pH values on degradation of BHA in PMS/Fe(VI). Experimental conditions: [BHA]_0_ = 100 μM, [PMS]_0_:[Fe(VI)]_0_:[BHA]_0_ = 100:1:1 and T = 25 °C.

The experiment of oxidative degradation of BHA by PMS alone was carried out under the conditions of [BHA]_0_ = 100 μM, pH = 8.0 and T = 25 °C. As shown in Figure 1a, when the proportion of PMS was constantly increased, the improvement in degradation rates was not significant enough. Within 30 min, when the ratio of [PMS]_0_:[BHA]_0_ was 10:1, 50:1, 100:1, 150:1 and 200:1, the degradation rates of BHA were 5.9%, 9.3%, 15.1%, 29.7% and 39.8%, respectively; this was because PMS did not readily generate more SO_4_^•−^ without the influence of external energy, which was consistent with the results from previous studies [38].

As shown in Figure 1b, at [PMS]_0_:[BHA]_0_ = 100:1, the degradation rate of BHA was only 15.1% in 30 min. In contrast, while the Fe(VI) process alone rapidly degraded 37.4% of the BHA in 30 s, the BHA was barely oxidized for the next 30 min. The effect on the degradation of BHA when PMS and Fe(VI) were involved in the reaction simultaneously was investigated. When [PMS]_0_:[Fe(VI)]_0_:[BHA]_0_ = 100:1:1, the degradation trend was essentially the same as for the Fe(VI) system alone, but the degradation rate was significantly faster, reaching 92.4%. The degradation efficiency of BHA using the PMS/Fe(VI) process was improved by 29.3% compared to that when the PMS system and Fe(VI) system were combined. Changes in the concentration of Fe(VI) were measured to explain why the addition of PMS to Fe(VI) increased the degradation of BHA, as shown in Figure 1c. It is clear that the presence of PMS accelerates the reduction in Fe(VI), suggesting that the addition of PMS promotes the rapid reaction of Fe(VI) with BHA. These results confirmed that the combined process of Fe(VI) and PMS had a stronger synergistic influence on the degradation of BHA.

### 3.2. Effect of pH on the Reaction

The pH value plays an essential role in the oxidation and degradation process in pollutants [39]. Thus, the initial pH of the reaction solutions was varied from 3.0 to 12.0 to examine its effect on the degradation of BHA. The experimental results are shown in Figure 1d. The degradation rate of BHA increased with an increasing pH value. The reaction degradation rate reached 100% when the pH value was greater than 11.0.

This result was a combination of multiple factors. First, the pK_a_ value of BHA in aqueous solution is 8.8. When the pH value is greater than 8.8, BHA presents in an ion state, which has a higher activity than when in a molecular state [40]. Second, Fe(VI) has three pK_a_ values in aqueous solution, 1.6, 3.5 and 7.3, corresponding to H_2_FeO_4_, HFeO_4_^−^, and FeO_4_^2−^, respectively (Equations (1)–(3)) [41]. With an increasing solution pH, Fe(VI) gradually changed from HFeO_4_^−^ under acidic conditions to FeO_4_^2−^ under alkaline conditions. Although the redox potential of Fe(VI) decreased from 2.2 V to 0.7 V, the stability increased and the self-decomposition rate decreased; then, the reaction of Fe(VI) with pollutants was more effective under alkaline conditions [42,43]. In addition, Fe^2+^ (generated through Fe(VI) decomposition) can react with PMS to produce more SO_4_^•−^ ((Equation (4)) [44]. However, under acidic conditions, excess H^+^ combines with HSO_5_^−^ to form hydrogen bonds, preventing this reaction from proceeding [30]. Due to the combined effects of the above reasons, the degradation rate of BHA showed a trend of pH-related variation.

H_3_FeO_4_^+^ ⇋ H^+^ + H_2_FeO_4_ (pK_a_ = 1.6) (1)

H_2_FeO_4_ ⇋ H^+^ + HFeO_4_^−^ (pK_a_ = 3.5) (2)

HFeO_4_^−^ ⇋ H^+^ + FeO_4_^2−^ (pK_a_ = 7.3) (3)

HSO_5_^−^ + Fe^2+^→SO_4_^•−^ + Fe^3+^ + OH^−^ (4)

### 3.3. Effect of Water Constituents

**Figure 2:**
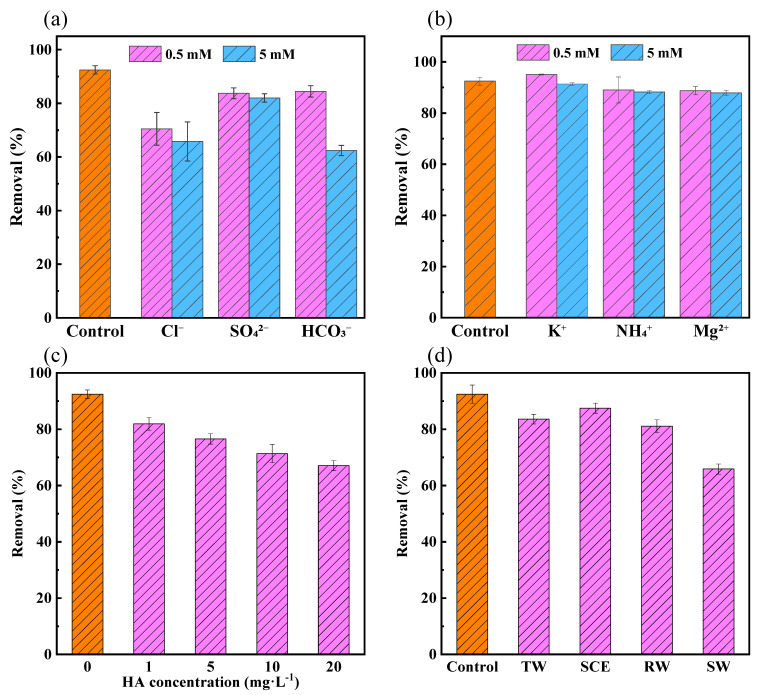
Effects of (**a**) anions, (**b**) cations, (**c**) humic acid and (**d**) four kinds of water matrices on the degradation of BHA in PMS/Fe(VI); note: tap water (TW), secondary clarifier effluent (SCE), river water (RW) and synthetic wastewater (SW). Experimental conditions: [BHA]_0_ = 100 μM, [PMS]_0_:[Fe(VI)]_0_:[BHA]_0_ = 100:1:1, pH = 8.0, T = 25 °C and reaction time = 30 min.

It has been reported that the degradation efficiency of pollutants may be affected by the presence of various inorganic ions in natural waters [45]. The effect of several common inorganic anion ions and cations in natural water on the degradation of BHA was investigated. Three anions (Cl^−^, SO_4_^2−^ and HCO_3_^−^) and three cations (K^+^, NH_4_^+^ and Mg^2+^) were included in the experiment (Figure 2a,b).

In previous studies, Fe(VI) degraded micropollutants, such as chlorophene, 4-tert-butylphenol and chlorpyrifos, and Cl^−^ had no significant effect on the Fe(VI) degradation of pollutants [43,45]. However, Cl^−^ can compete with contaminants for ^•^OH and SO_4_^•−^ in PMS/Fe(VI) systems [46,47]. Therefore, as the concentration of Cl^−^ increases, the BHA degradation rate of the reaction decreases. Compared with Cl^−^, the inhibition was more pronounced with an increasing HCO_3_^−^ concentration. This is due to the ability of HCO_3_^−^ to quench ^•^OH and SO_4_^•−^ [48]. And HCO_3_^−^ has a certain inhibitory effect on the oxidation of Fe (VI) [49]. SO_4_^2−^, K^+^, NH_4_^+^ and Mg^2+^ had almost no influence on the degradation rate of BHA. Similar results were also reported in previous studies [28,50]. This study also tested the effects of humic acid at different concentrations on the degradation of BHA by PMS/Fe(VI). As shown in Figure 2c, the addition of HA inhibited the efficiency of the PMS/Fe(VI) degradation of BHA, and the inhibition effect was more obvious at high concentrations of HA. This is due to the ability of HA to competitively consume the oxidant, resulting in a decrease in the removal of the target pollutant [51].

### 3.4. Effect of Water Matrices

In addition to ions and HA, the composition of natural waters are very complex. It is crucial to evaluate the feasibility of using PMS/Fe(VI) for BHA removal in real natural waters. The degradation of BHA by PMS/Fe(VI) in four different water matrices (tap water (TW), secondary clarifier effluent (SCE), river water (RW) and synthetic wastewater (SW)) is shown in Figure 2d. TW, SCE and RW had a slight inhibitory effect on the degradation of BHA in the PMS/Fe (VI) system. The lowest degradation rate of BHA was observed in SW. This is due to the fact that the highest content of Cl^−^ and TOC in SW will compete with the target compounds to consume the oxidant, which is consistent with the findings of previous research studies [35]. Therefore, the amount of PMS/Fe(VI) needs to be increased in organic-rich wastewater. Overall, PMS/Fe(VI) can be used as an effective technique to degrade coexisting BHA in natural waters.

### 3.5. Identification of Main Reactive Species

**Figure 3:**
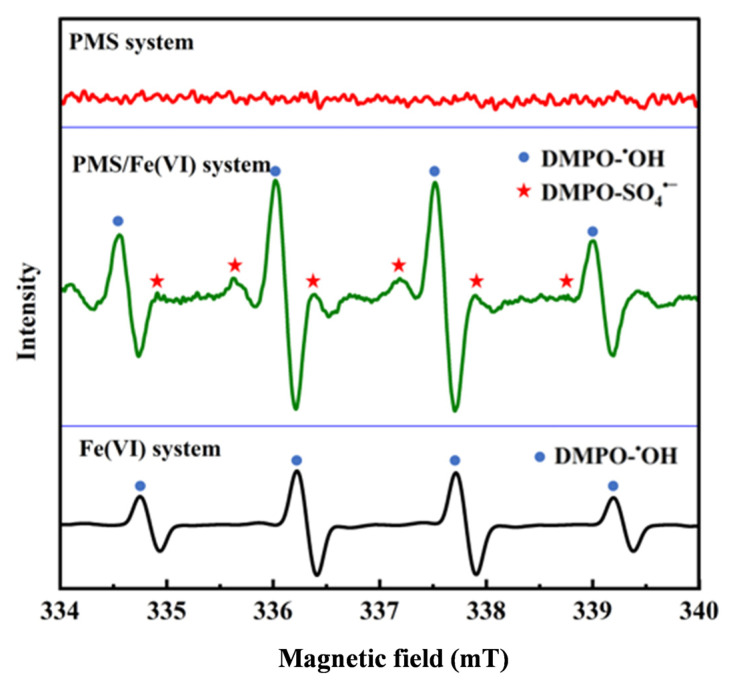
EPR spectra of hydroxyl and sulfate radicals trapped by DMPO in deionized water.

According to the results of previous studies, in the PMS system, SO_4_^•−^ and ^•^OH were the main species, while in the Fe(VI) system, ^•^OH was produced by the self-decay of Fe(VI) [52,53]. Potentially active species were identified using EPR techniques. As shown in Figure 3, adding DMPO to the PMS system alone did not result in spikes. In contrast, a distinct quartet spectrum with an intensity ratio of 1:2:2:1 and a splitting constant of αN = αH = 14.9 G was present in the PMS/Fe(VI) and Fe(VI) systems, which was considered to be the signaling peak of the hydroxyl radical. In addition, the intensity of the DMPO-^•^OH peak in the PMS/Fe(VI) system was significantly higher than that in the Fe(VI) system, which also indicated a synergistic effect between PMS and Fe(VI). At the same time, SO_4_^•−^ was also produced in the PMS/Fe(VI) oxidation system, with a center of symmetry of g = 2.005 and an intensity ratio of 1:1:1:1:1:1 for the six-line signals [54]. Therefore, the accelerated degradation of BHA in the PMS/Fe(VI) oxidation system was attributed to the synergistic effect between PMS and Fe(VI) and the generation of reactive radicals.

### 3.6. The Reaction Pathway

**Figure 4:**
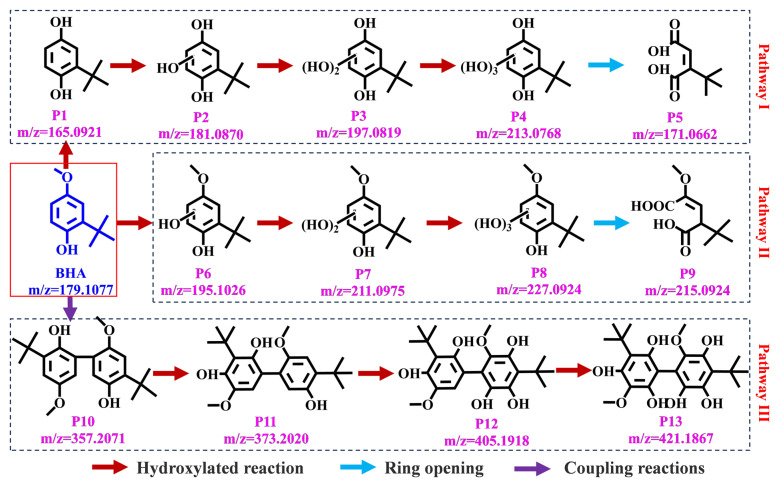
The proposed reaction pathway of BHA oxidation by PMS and PMS/Fe(VI).

By using LC/MS-MS, it was found that the reaction intermediate species of BHA during degradation in the single-PMS system and PMS/Fe(VI) system were the same. A total of 13 products (P1–P13) were identified, and the detailed mass spectral information and MS/MS fragmentation patterns are shown in Table S2 and Figure S1. The differences between the calculated and measured masses never exceeded 5.0 ppm, which confirmed that the proposed molecular formulae were highly reliable. Based on these identified products, as shown in Figure 4, three different transformation pathways for the oxidative degradation of BHA by PMS and PMS/Fe(VI) were proposed, which mainly included hydroxylated, ring-opening and coupling reactions.

In pathway I, ^•^OH replaced the methoxy group of BHA to produce the hydroxylated product P1 and methyl alcohol. In pathway II, ^•^OH attacked the aromatic C atom of BHA to generate a hydroxylation product P6. P1 and P6 continued the hydroxylation reaction under the attack of ^•^OH to form polyhydroxylated products (P2→P3→P4 and P7→P8). Furthermore, in addition to the ^•^OH attack, these polyhydroxylated products can also be generated by a mechanism of singlet oxygen transfer via Fe(VI) oxidation [34]. It has been reported that two adjacent or opposite hydroxyl groups on the benzene ring can be easily oxidized to form ketone groups [52]. Thus, the hydroxylation products P1–P4 and P6–P8 were oxidized into quinone intermediates. These intermediates underwent a ring-opening reaction to produce the carboxylic acid intermediates P5 and P9.

Another important pathway for the oxidation of phenol by Fe(VI) and PMS was the coupling reaction. Phenolic compounds are susceptible to the formation of phenoxy radicals by chemical oxidizers through hydrogen extraction or single-electron transfer [55]. Fe(VI) and PMS attack the electron-rich phenolic groups of phenol to generate the corresponding phenoxy radicals [45,50]. These unpaired-electron phenoxy radicals are not stable and tend to couple to each other, eventually forming stabilized products with C-C or C-O-C bonds. The polymerization product P10 was observed during the degradation of BHA by single PMS and PMS/Fe(VI). P10 was attacked by ^•^OH to continue the hydroxylation reaction to form polyhydroxylated polymerization products (P11→P12→P13). In addition, DFT theory was used to calculate the potential attack sites of BHA, and a detailed analysis was conducted, the results of which are presented in Text S3 and Figure S2. Compared with Fe(VI) and SO_4_^•−^, the activation energy of the ^•^OH attack on BHA is lower, and the corresponding hydroxylation products are easily generated. This was consistent with the degradation pathways which showed that the degradation intermediates were predominantly hydroxylated products.

### 3.7. Toxicity Evaluation

**Figure 5:**
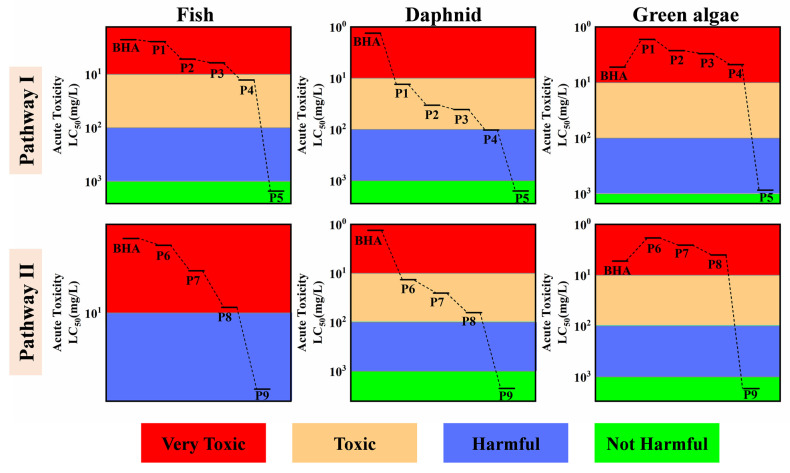
PMS/Fe(VI) oxidation BHA reaction intermediates’ toxicity evaluation heatmap.

It is critical to give attention to the ecological risks in water bodies after treatment. BHA degrades into the same product types within PMS and PMS/Fe(VI) systems; however, the abundance of the products varies greatly between the two systems. Therefore, a comparison of the changes in product abundance and toxicity in the two systems is extremely necessary. The acute toxicity of the products of each pathway was predicted by the ECOSAR software, as shown in Figure 5. BHA was found to be extremely toxic to fish, Daphnia and green algae. The toxicity of the transformation intermediates showed an overall decreasing trend compared to the toxicity of the parent compounds. In pathways I and II, P1–P4 and P6–P8 remained at toxic levels for fish, Daphnia and algae. However, the aquatic toxicity to fish and Daphnia was generally reduced. The end products of both pathways, P5 and P9, were virtually nontoxic. The molecular weight of the generated polymerization coupling products (P10–P13) is high, and therefore, accurate toxicity predictions cannot be obtained by ECOSAR. According to the available studies, polymerization products of large molecules have low solubility and low bioavailability properties [56]. As a result, they can be separated from water by simple physical means, which facilitated the control of environmental risks.

**Figure 6:**
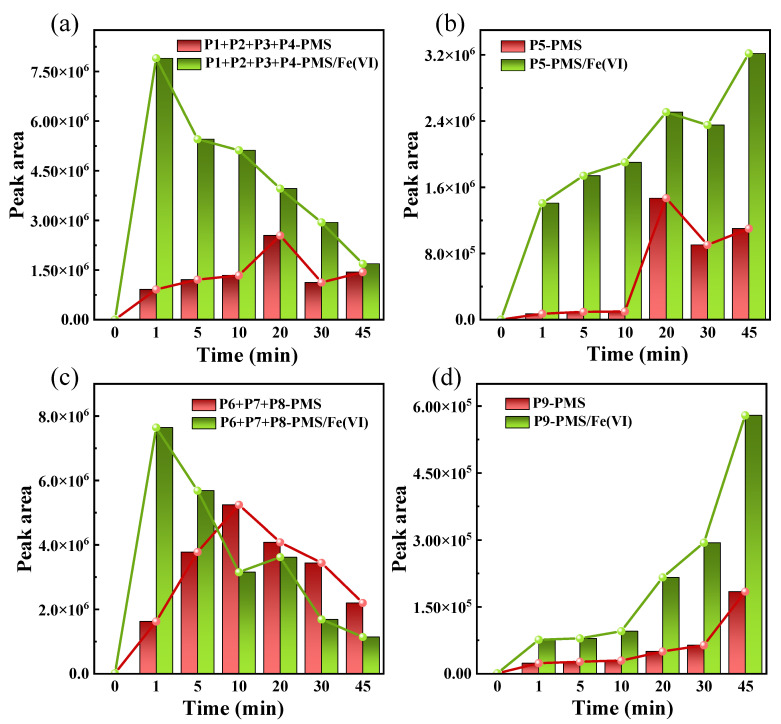
Changes in abundance of (**a**) P1-P4, (**b**) P5, (**c**) P6-P8 and (**d**) P9 of BHA oxidation by PMS/Fe(VI) and PMS alone. PMS/Fe(VI) oxidation experiment: [BHA]_0_ = 100 μM, [PMS]_0_:[Fe(VI)]_0_:[BHA]_0_ = 100:1:1, pH = 8.0, T = 25 °C. PMS oxidation experiment: [BHA]_0_ = 100 μM, [PMS]_0_:[BHA]_0_ = 200:1, T = 50 °C.

In the degradation of BHA, the presence of intermediates cannot be ignored. Analyzing the toxicity of a single product is not representative of assessing changes in the toxicity of the entire reaction system. It is more informative to assess the toxicity of the reaction system in terms of changes in the abundance of all products. In pathway I, although the P1–P4 toxicity level was relatively high, the overall concentration decreased with reaction time in the PMS/Fe(VI) system, and the peak area at the end of the reaction was similar to that of the PMS system (Figure 6a). As the end product of pathway I, P5 was nontoxic, and the peak area of the PMS/Fe(VI) system was significantly higher than that of the PMS system (Figure 6b), therefore, the toxicity of PMS/Fe(VI) in pathway I was lower than that of PMS.

In pathway II, the P6–P8 toxicity level remained relatively high, but in the PMS/Fe(VI) system, the peak area decreased with reaction time (Figure 6c). In contrast, as the reaction progressed, the peak areas of P6–P8 in the PMS system showed a tendency to increase and then decrease, and eventually, the peak area of P6–P8 in the PMS/Fe(VI) system was half of that in the PMS system. P9 was the final product of pathway II and was harmless to three aquatic organisms. The PMS/Fe(VI) system produced more P9 during the reaction (approximately 3 times more than the PMS system) (Figure 6d).

In summary, the toxicity of the combined PMS/Fe(VI) system was lower than that of the single-PMS system in all cases, suggesting that the combined approach can reduce the toxicity during BHA degradation. Overall, this PMS/Fe(VI) technology can be a promising and safe method for treating contaminants.

## 4. Conclusions

The results showed that PMS/Fe(VI) had a synergistic effect on the degradation of BHA compared to a single-PMS system. When T = 25 °C, the initial pH was 8.0 and the initial BHA concentration was 100 μM, the initial concentration ratio of [PMS]_0_:[Fe(VI)]_0_:[BHA]_0_ was 100:1:1, and the degradation rate could reach 92.4% within 30 min. In addition, the degradation efficiency of BHA increased with increasing solution pH in the PMS/Fe (VI) system. K^+^, NH_4_^+^ and Mg^2+^ had a promoting effect on the reaction. Based on the intermediates identified with LC-QTOF-MS, the oxidation of BHA by PMS/Fe(VI) included hydroxylation, ring-opening and coupling reactions. The main active species of the reaction system were identified with EPR tests, and this result was correlated with theoretical calculations. Based on the results of DFT calculations, it can be seen that BHA was more susceptible to attack by hydroxyl radicals, generating hydroxylation products. A comprehensive comparison of the toxicity was performed, and the PMS/Fe(VI) system reduced the environmental risk compared to the single-PMS system. Importantly, the results of these studies may provide useful information for the removal of pollutants in real water bodies and may also deepen the understanding of the behavior of the PMS/Fe oxidation system in transforming pollutants. The above results indicate that the combined treatment of PMS and Fe(VI) is a safe and efficient route for BHA degradation. This study provides more favorable results that support the use of this combined oxidation method for water treatment.

## Data Availability

Data will be made available on request.

## Supplementary Materials

The following supporting information can be downloaded at: https://www.mdpi.com/article/10.3390/toxics12010054/s1, Text S1: Analytical method of HPLC; Text S2: Analytical method of LC-MS; Text S3: Theoretical Analysis; Table S1: Water quality parameters of natural water samples*; Table S2: Accurate mass measurements of the degradation products of BHA during oxidation as determined by LC- MS. Noted that Rt means retention time; Figure S1: Product ion spectra of reaction intermediates during PMS/Fe(VI) oxidation of BHA; Figure S2: Energy barriers for the reaction of (a) Fe(VI), (b) SO_4_^•−^ and (c) ^•^OH with BHA calculated at the b3lyp/6-311 g(d,p) level.
