# Supplementary material for: Degradation of Butylated Hydroxyanisole by the Combined Use of Peroxymonosulfate and Ferrate(VI): Reaction Kinetics, Mechanism and Toxicity Evaluation
Source: Toxics. 2024 Jan 10;12(1):54. doi: 10.3390/toxics12010054 (PMC10818440; doi:10.3390/toxics12010054)
Supplement: Supplementary file 1 [file toxics-12-00054-s001.zip › toxics-2774549-supplementary.pdf]

Supporting Information

Degradation of Butylated Hydroxyanisole by the Combined Use of Peroxymonosulfate and Ferrate(VI): Reaction Kinetics, Mechanism and Toxicity Evaluation

Peiduan Shi [1,†], Xin Yue [1,†], Xiaolei Teng [1,*], Ruijuan Qu [1], Ahmed Rady [2], Saleh Maodaa [2], Ahmed A. Allam [3], Zunyao Wang [1] and Zongli Huo [4,*]

[1] State Key Laboratory of Pollution Control and Resources Reuse, School of the Environment, Nanjing University, Nanjing 210023, China; peiduan_shi@163.com (P.S.); point_xinyue@163.com (X.Y.); quruijuan0404@nju.edu.cn (R.Q.); wangzy@nju.edu.cn (Z.W.)

[2] Department of Zoology, College of Science, King Saud University, P.O. Box 2455, Riyadh 11451, Saudi Arabia; jajarem@ksu.edu.sa (A.R.); maodaa_28@yahoo.com (S.M.)

[3] Department of Zoology, Faculty of Science, Beni-Suef University, Beni-Suef 65211, Egypt; allam1081981@yahoo.com

[4] Jiangsu Provincial Center for Disease Control and Prevention, No. 172 Jiangsu Road, Nanjing 210009, China

* Correspondence: tengxiaolei@smail.nju.edu.cn (X.T.); huozl@jscdc.cn (Z.H.)

† These authors contributed equally to this work.

Text S1 Analytical method of HPLC.

The BHA content was detected with the help of a Flexar PerkinElmer liquid chromatograph (USA) under a UV detector.The liquid phase determination parameters were as follows: mobile phase consisted of 60% methanol and 40% 0.3% formic acid water, C18 column(150 mm × 4.6 mm, 5 μm), detection wavelength 280 nm, injection volume 50 μL, flow rate 0.5 $mL·min^{-1}$.The column temperature was 30 ℃, and the total running time was 5 min.

Text S2 Analytical method of LC-MS.

Chromatographic separation was conducted on a S4 Thermo BDS Hypersil C18 column (2.1 mm × 100 mm, particle size 2.4 μm) (Thermo Fisher Scientific, Waltham, MA) at 30 °C. The mobile phase consisted of 0.1% formic acid in water (A) and methanol (B), and the flow rate was 200 μL min-1. The following elution gradient program was used: 10% B (0 ~ 4 min), 10 ~ 60% B (4 ~ 4.5 min, held for 3min), 60 ~ 80% B (7.5 ~ 8 min, held for 3min), 80 ~ 90% B (11 ~ 11.5 min, held for 3.5 min), 90 ~ 100% B (15 ~ 15.5 min, held for 9.5 min), and 100 ~ 10% B (25 ~ 25.5 min, held for 9.5 min). The MS spectrometer was operated with electrospray ionization (ESI) source in negative ion mode to record mass spectra (m/z 60 ~ 1500) under the following conditions: ionspray voltage floating, -4500 V; temperature, 550 °C; ion source gas 1, 55 psi; ion source gas 2, 55 psi; curtain gas, 35 psi; declustering potential, -80 V; collision energy, -10 V . the analysis. Following the MS analysis, the parent ions of possible transformation products were individually selected for MS/MS analysis, and the fragmentation was optimized by varying collision energy from 25 to 65 eV. During the analysis, nitrogen gas was used throughout, and the accuracy of the MS and MS/MS data was ensured by a calibration error of < 3 ppm during external mass calibration with APCI negative calibration solution. The high-resolution LC-MS data were acquired with Analyst TF software (V ersion 1.6, AB Sciex) and processed using PeakView software (V ersion 1.2, AB Sciex).

Text S3 Theoretical Analysis

Theoretical calculations as a useful addition to experimental work have been applied in this work to reveal the transformation mechanisms of various organic pollutants. As discussed above, the active species $SO_4^{\bullet-}$ and $^{\bullet}OH$ in the PMS/Fe(VI) system, in addition, Fe(VI) can oxidize pollutants by means of oxygen transfer. Interestingly, among the degradation products, the detected products were mainly hydroxylation products and no sulfate addition products were detected. Therefore, in order to determine the most probable structure of the reaction intermediate, DFT method was used to calculate the transition states (TSs) at different sites of Fe(VI), $SO_4^{\bullet-}$ and $^{\bullet}OH$ attack on BHA. Notably, the higher the activation energy, the less likely the reaction is to occur. As shown in Fig. S2, three sites C(3), C(5) and C(6) were calculated. The Fe(VI) attack energy barriers for TS_{3C}, TS_{6C}, and TS_{5C} were 90.99, 91.42 and 125.10 kJ/mol, respectively. The energy barriers of TS_{3C}, TS_{6C} and TS_{5C} were 87.19, 97.99 and 139.62 kJ/mol, respectively, for the $SO_4^{\bullet-}$ attacked BHA, as shown in Fig. S2b. While in $^{\bullet}OH$ attacking BHA process, the energy barriers of TS_{3C}, TS_{5C} and TS_{6C} were 4.63, 9.64 and 9.76 kJ/mol, respectively (Fig. S2c). Therefore, compared with Fe(VI) and $SO_4^{\bullet-}$, $^{\bullet}OH$ is more prone to attack C(3), C(5) and C(6) sites of BHA to generate corresponding hydroxylation products.

Table S1. Water quality parameters of natural water samples*.

Parameter	Unit	Synthetic wastewater	River water	Secondary clarifier effluent	Tap water
pH	--	7.82	7.47	7.27	6.95
TOC	mg/L	39.68	19.98	22.08	3.15
Cl^-	mg/L	1295.77	42.15	269.63	14.27
NO_3^-	mg/L	0.32	58.93	52.76	9.61
SO_4^{2-}	mg/L	ND	144.19	139.28	38.58
Na^+	mg/L	671.91	8.10	60.40	4.79
K^+	mg/L	0.54	4.67	11.52	2.44
Mg^{2+}	mg/L	6.22	7.60	17.44	5.14
Ca^{2+}	mg/L	4.56	6.29	4.91	3.04
Cu^{2+}	µg/L	6.21	8.76	10.10	123.41

* Total organic carbon (TOC) was measured by a TOC analyzer (OI - 1030D, USA). The concentrations of anions and metal ions were detected by IC 1000 and ICP - MS, respectively.

ND: Not detected.

Table S2. Accurate mass measurements of the degradation products of BHA during oxidation as determined by LC- MS. Noted that R_t means retention time.

Compound	R_t/min	Molecular formula	Calculated mass (m/z)	Experimental mass(m/z)	Error(ppm)
BHA	10.522	$C_{11}H_{16}O_2$	179.1077	179.1075	-1.12
	10.860	$C_{11}H_{16}O_2$	179.1077	179.1076	-0.56
P1	9.933	$C_{10}H_{14}O_2$	165.0921	165.0913	-4.85
	10.066	$C_{10}H_{14}O_2$	165.0921	165.0918	-1.82
	10.430	$C_{10}H_{14}O_2$	165.0921	165.0919	-1.21
P2	6.427	$C_{10}H_{14}O_3$	181.0870	181.0868	-1.10
	7.023	$C_{10}H_{14}O_3$	181.0870	181.0868	-1.10
	7.871	$C_{10}H_{14}O_3$	181.0870	181.0866	-2.21
	8.563	$C_{10}H_{14}O_3$	181.0870	181.0869	-0.55
	8.866	$C_{10}H_{14}O_3$	181.0870	181.0867	-1.66
P3	8.577	$C_{10}H_{14}O_4$	197.0819	197.0818	-0.51
	8.813	$C_{10}H_{14}O_4$	197.0819	197.0821	1.01
P4	6.244	$C_{10}H_{14}O_5$	213.0768	213.0768	0.00
	6.943	$C_{10}H_{14}O_5$	213.0768	213.0768	0.00
	7.469	$C_{10}H_{14}O_5$	213.0768	213.0769	0.47
	7.944	$C_{10}H_{14}O_5$	213.0768	213.0768	0.00
P5	7.514	$C_8H_{12}O_4$	171.0662	171.0662	0.00
P6	10.380	$C_{11}H_{16}O_3$	195.1026	195.1024	-1.03
P7	8.286	$C_{11}H_{16}O_4$	211.0975	211.0975	0.00
	8.567	$C_{11}H_{16}O_4$	211.0975	211.0974	-0.47
	10.061	$C_{11}H_{16}O_4$	211.0975	211.0976	0.47
P8	7.837	$C_{11}H_{16}O_5$	227.0924	227.0926	0.88
	9.001	$C_{11}H_{16}O_5$	227.0924	227.0924	0.00
P9	6.365	$C_{10}H_{16}O_5$	215.0924	215.0922	-0.93
	8.094	$C_{10}H_{16}O_5$	215.0924	215.0926	0.93
	8.413	$C_{10}H_{16}O_5$	215.0924	215.0921	-1.39
P10	13.000	$C_{22}H_{30}O_4$	357.2071	357.2066	-1.40
P11	12.491	$C_{22}H_{30}O_5$	373.2020	373.2016	-1.07
	12.647	$C_{22}H_{30}O_5$	373.2020	373.2024	1.07
P12	10.994	$C_{22}H_{30}O_7$	405.1918	405.1919	0.25
P13	10.810	$C_{22}H_{30}O_8$	421.1867	421.1882	3.56
	11.016	$C_{22}H_{30}O_8$	421.1867	421.1873	1.42
	11.167	$C_{22}H_{30}O_8$	421.1867	421.1871	0.95
	11.332	$C_{22}H_{30}O_8$	421.1867	421.1871	0.95
	11.552	$C_{22}H_{30}O_8$	421.1867	421.1869	0.47

P1 m/z=165.0921

Spectrum from 20230619-SPD-ii.wiff2 (sample 1) - 20230619-SPD-165.0921, -TO...of 165.1 (40 - 1500) from 0.825 to 4.596 min Precursor: 165.1 Da, CE: -15.0

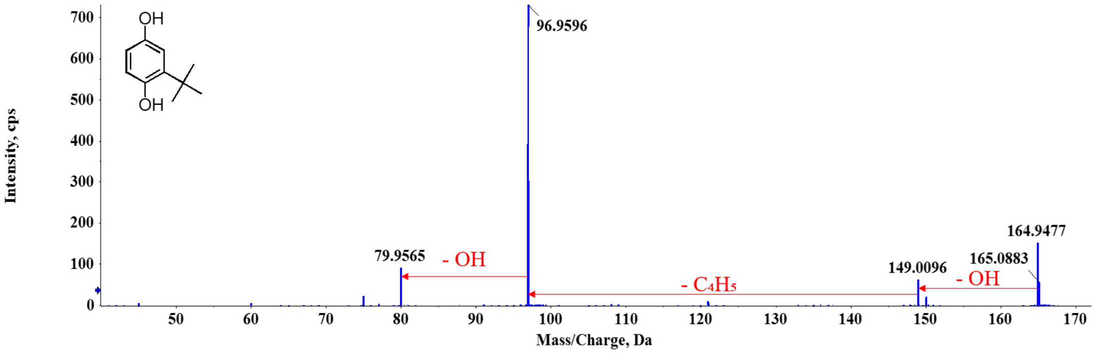

P2 m/z=181.0870

Spectrum from 20230619-SPD-ii.wiff2 (sample 2) - 20230619-SPD-181.0870, -TOF MSMS of 181.1 (40 - 1500) from 0.886 min Precursor: 181.1 Da, CE: -20.0

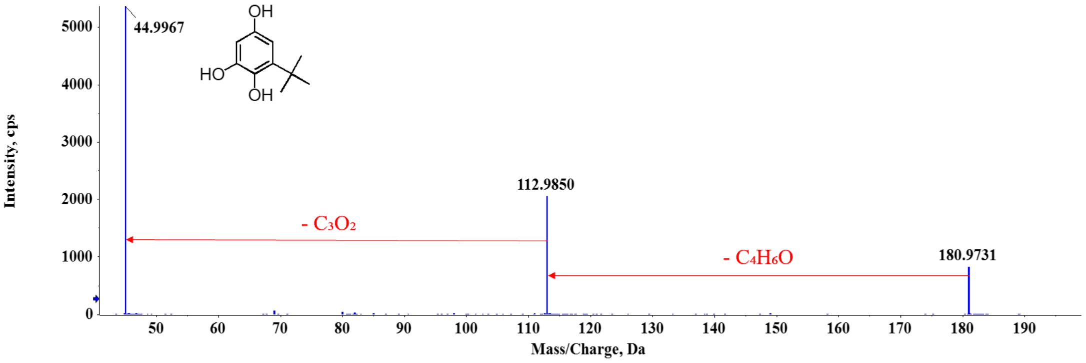

P3 m/z=197.0819

Spectrum from 20230619-SPD-ii.wiff2 (sample 5) - 20230619-SPD-197.0819, -TOF MSMS of 197.1 (40 - 1500) from 1.024 min Precursor: 197.1 Da, CE: -20.0

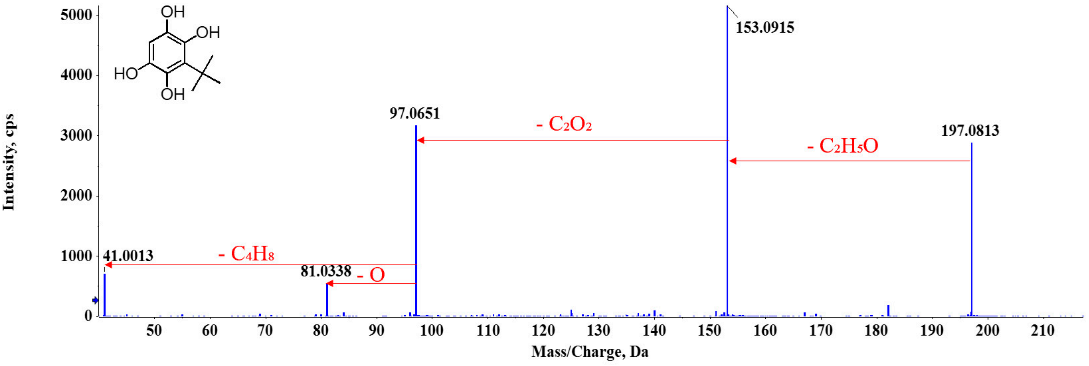

P4 m/z=213.0768

Spectrum from 20230619-SPD-ii.wiff2 (sample 3) - 20230619-SPD-213.0768, -TOF MSMS of 213.1 (40 - 1500) from 1.069 min Precursor: 213.1 Da, CE: -20.0

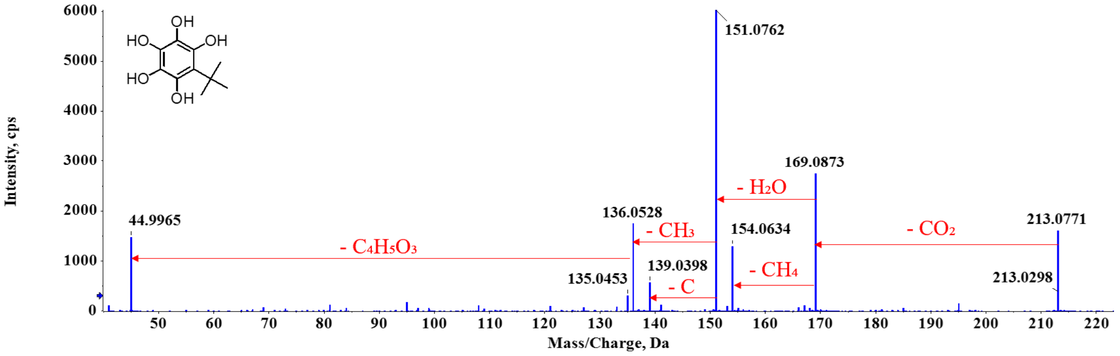

P5 m/z=171.0662

Spectrum from 20230619-SPD-ii.wiff2 (sample 22) - 20230619-SPD-171.0662, -TOF MSMS of 171.1 (40 - 1500) from 1.174 min Precursor: 171.1 Da, CE: -20.0

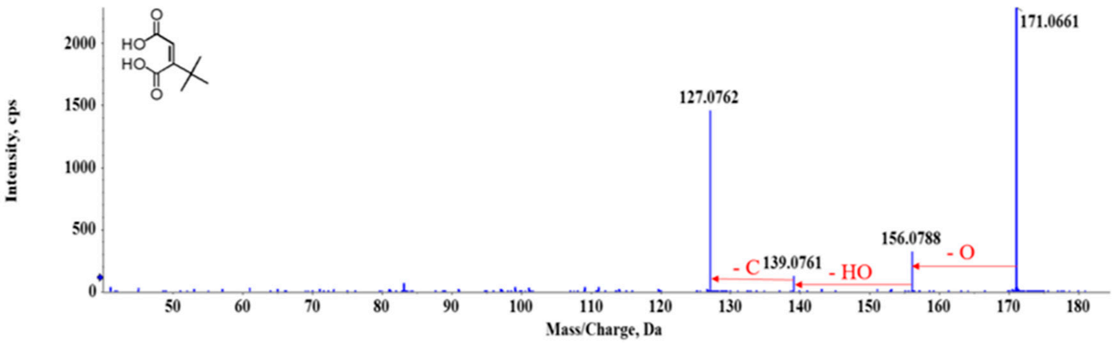

P6 m/z=195.1026

Spectrum from 20230619-SPD-ii.wiff2 (sample 7) - 20230619-SPD-195.1026, -TOF MSMS of 195.1 (40 - 1500) from 1.040 min Precursor: 195.1 Da, CE: -20.0

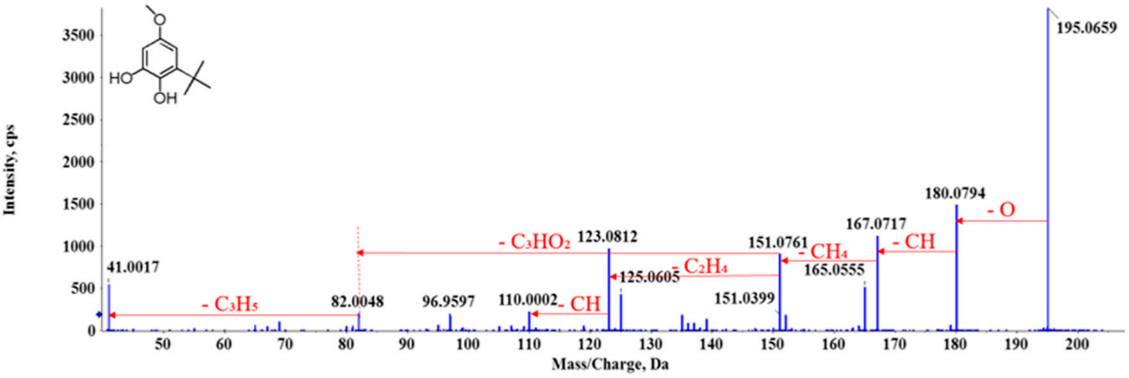

P7 m/z=211.0975

Spectrum from 20230619-SPD-ii.wiff2 (sample 16) - 20230619-SPD-211.0975, -TOF MSMS of 211.1 (40 - 1500) from 1.069 min Precursor: 211.1 Da, CE: -20.0

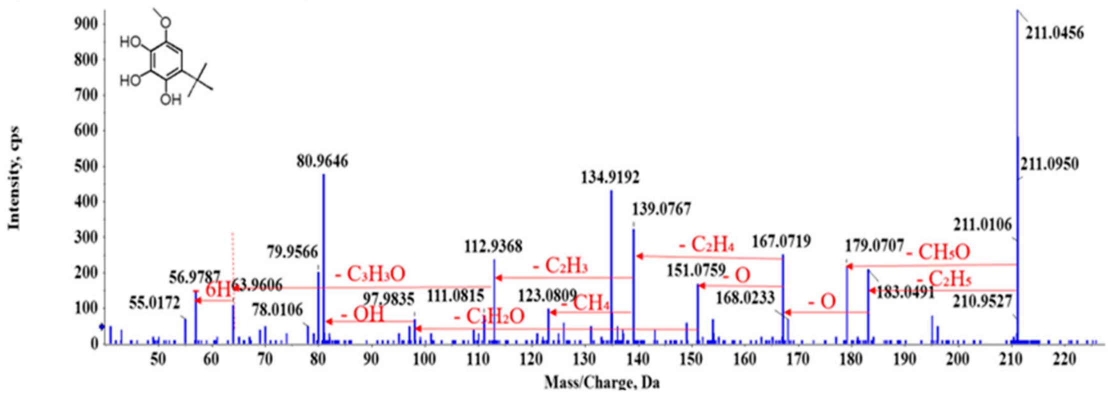

P8 m/z=227.0924

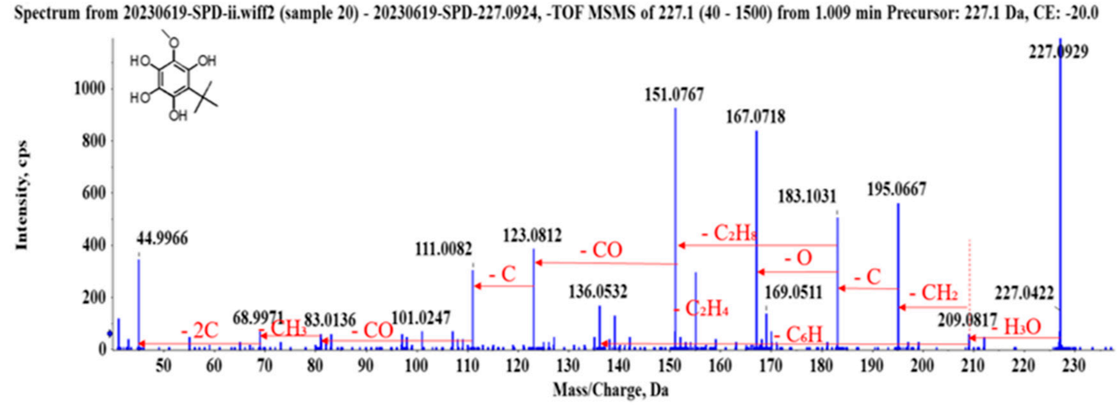

P9 m/z=215.0924

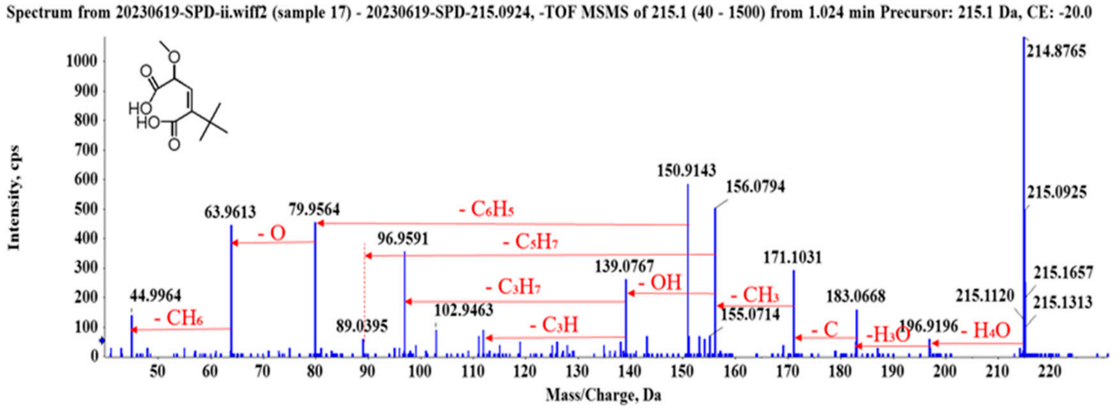

P10 m/z=357.2071

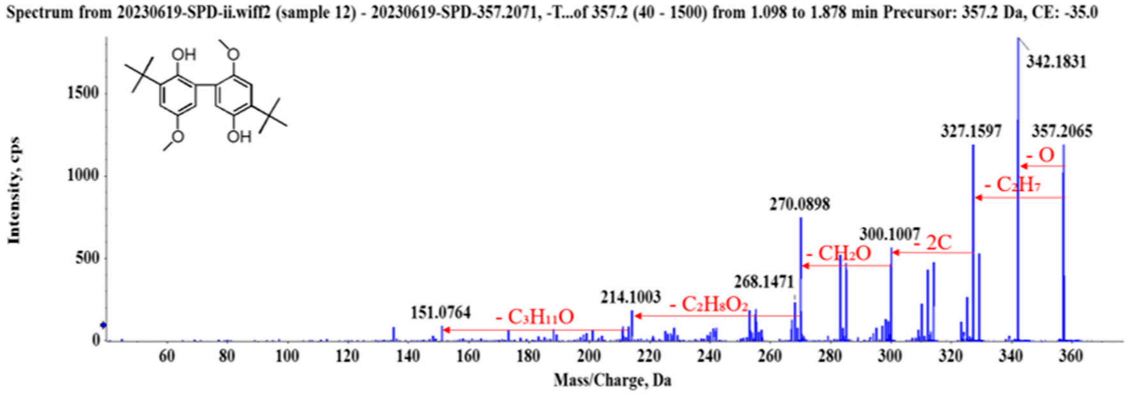

P11 m/z=373.2020

Spectrum from 20230619-SPD-ii.wiff2 (sample 18) - 20230619-SPD-373.2020, -TOF MSMS of 373.2 (40 - 1500) from 1.195 min Precursor: 373.2 Da, CE: -40.0

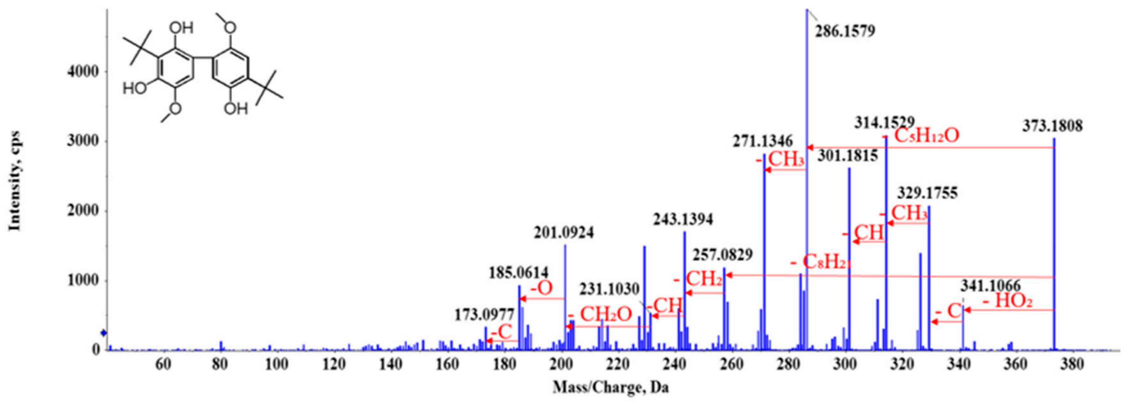

P12 m/z=405.1918

Spectrum from 20230619-SPD-ii.wiff2 (sample 19) - 20230619-SPD-405.1918, -TOF MSMS of 405.2 (40 - 1500) from 1.151 min Precursor: 405.2 Da, CE: -40.0

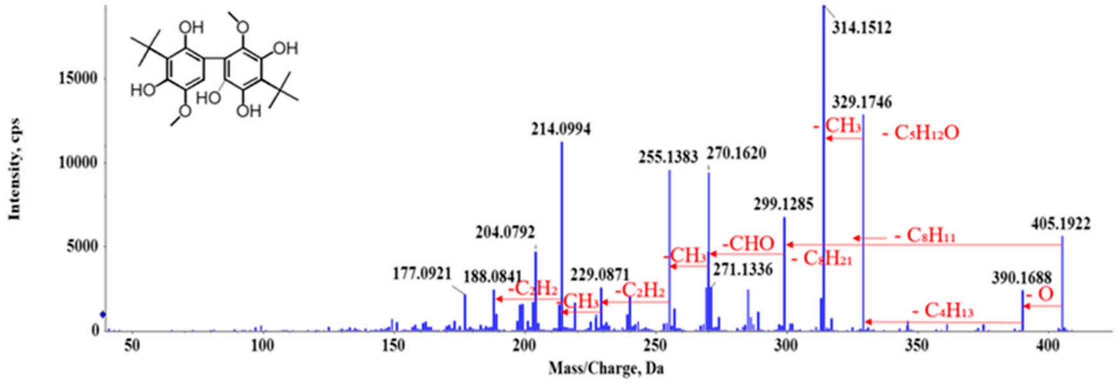

P13 m/z=421.1867

Spectrum from 20230619-SPD-ii.wiff2 (sample 13) - 20230619-SPD-421.1867, -T...of 421.2 (40 - 1500) from 0.883 to 1.331 min Precursor: 421.2 Da, CE: -40.0

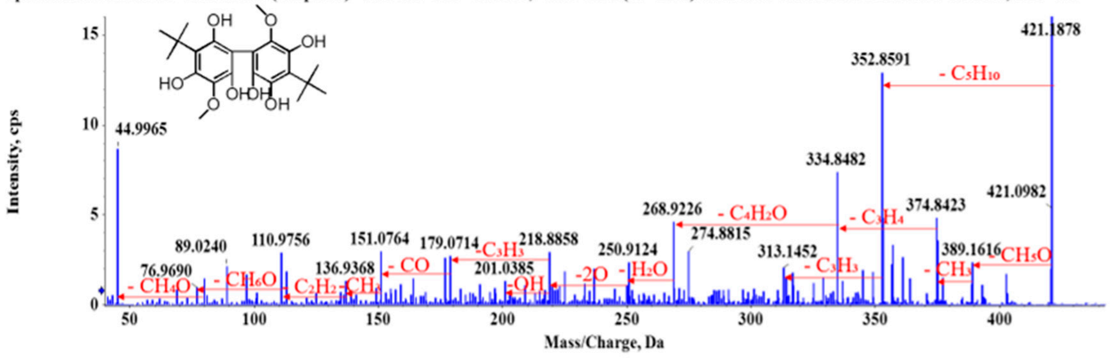

Figure S1. Product ion spectra of reaction intermediates during PMS/Fe(VI) oxidation of BHA. Experimental conditions: Experimental conditions: $[BHA]_0$ = 0.1 mmol/L, $[PMS]_0$:$[Fe(VI)]_0$:$[BHA]_0$ = 100:1:1, pH = 8.0, T = 25 °C.

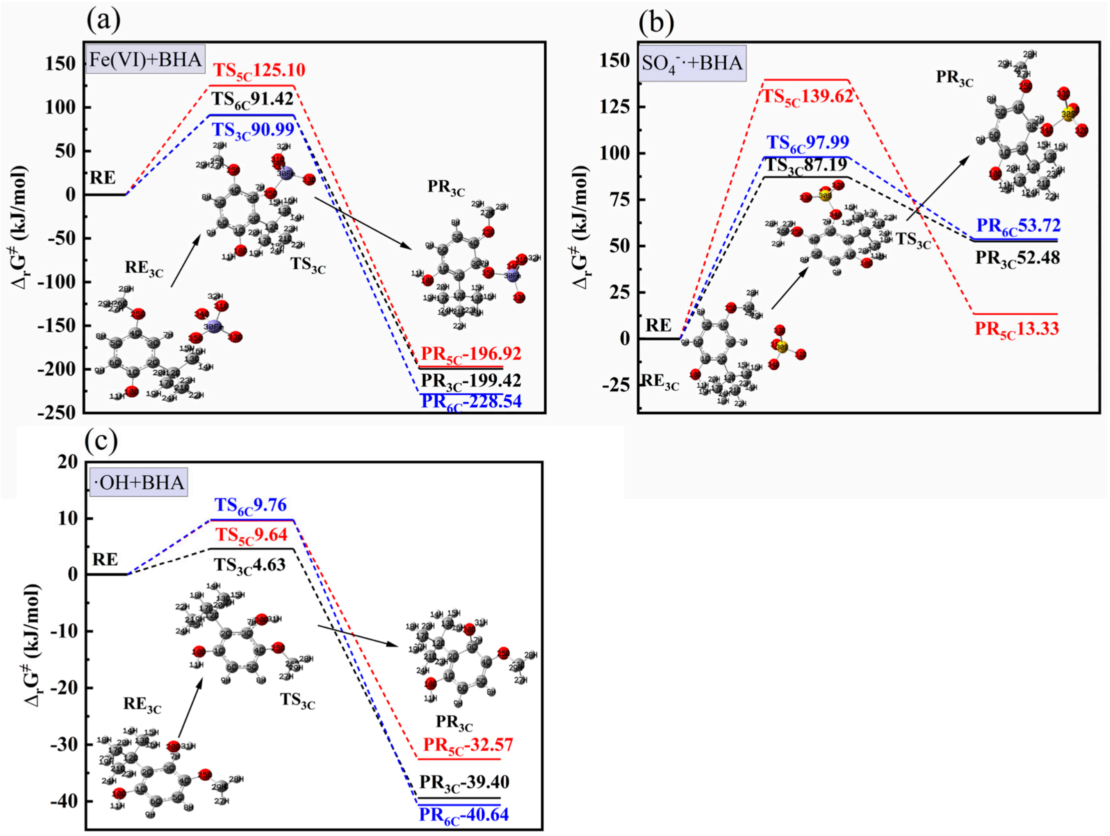

Figure S2. Energy barriers for the reaction of (a) Fe(VI), (b) $SO_4^{\bullet-}$ and (c) $^{\bullet}OH$ with BHA calculated at the b3lyp/6-311 g(d,p) level.
